# Systematic exploration of guide-tree topology effects for small protein alignments

**DOI:** 10.1186/1471-2105-15-338

**Published:** 2014-10-04

**Authors:** Fabian Sievers, Graham M Hughes, Desmond G Higgins

**Affiliations:** University College Dublin, Conway Institute, Dublin, Ireland

**Keywords:** Multiple sequence alignment, Guide-tree topology, Alignment accuracy, Benchmarking

## Abstract

**Background:**

Guide-trees are used as part of an essential heuristic to enable the calculation of multiple sequence alignments. They have been the focus of much method development but there has been little effort at determining systematically, which guide-trees, if any, give the best alignments. Some guide-tree construction schemes are based on pair-wise distances amongst unaligned sequences. Others try to emulate an underlying evolutionary tree and involve various iteration methods.

**Results:**

We explore all possible guide-trees for a set of protein alignments of up to eight sequences. We find that pairwise distance based default guide-trees sometimes outperform evolutionary guide-trees, as measured by structure derived reference alignments. However, default guide-trees fall way short of the optimum attainable scores. On average chained guide-trees perform better than balanced ones but are not better than default guide-trees for small alignments.

**Conclusions:**

Alignment methods that use Consistency or hidden Markov models to make alignments are less susceptible to sub-optimal guide-trees than simpler methods, that basically use conventional sequence alignment between profiles. The latter appear to be affected positively by evolutionary based guide-trees for difficult alignments and negatively for easy alignments. One phylogeny aware alignment program can strongly discriminate between good and bad guide-trees. The results for randomly chained guide-trees improve with the number of sequences.

**Electronic supplementary material:**

The online version of this article (doi:10.1186/1471-2105-15-338) contains supplementary material, which is available to authorized users.

## Background

Multiple Sequence Alignments (MSAs) are an integral part of many bioinformatics analyses. From an evolutionary perspective, MSAs can be considered as attempts at arranging sequences in such a way that homologous residues occupy the same columns. This will necessitate the introduction of gaps into some or all of the sequences. A good alignment is found by searching for one that attains a favourable score by penalising the introduction of gaps and substitution of residues. Given a scoring function it is possible to determine the optimum alignment of two sequences using dynamic programming [1]. However, as time and memory requirements for determining the optimum alignment grow exponentially with the number of sequences, an exact solution is not feasible for more than a few sequences. This is why the ‘progressive alignment’ heuristic was developed [2]. In progressive alignment, initially only pairs of sequences are aligned, producing so-called profiles, which are then in turn aligned in a pair-wise fashion with other sequences or ever growing profiles. The order in which sequences and profiles are aligned is determined by a so-called ‘guide-tree’ [3]. A shortcoming of progressive alignment is that an arrangement of residues that was determined early in the MSA cannot be changed at a later stage [2]. Consequently the guide-tree has an effect on the quality of the final alignment, and there are different strategies of constructing such a guide-tree. A common strategy [4–6] is to determine pair-wise distances amongst the sequences and to construct from these distances either a UPGMA [7] or neighbour joining [8] tree. In another strategy [9] the guide-tree is made to resemble the evolutionary tree that is imputed to have given rise to the sequences. Recently we have demonstrated [10] that randomly labeled chained guide-trees produce good alignments for very large numbers of protein sequences. This approach is similar to early MSA strategies [11, 12] where sequences were simply added to a growing MSA, one by one. It is also similar to how alignments are generated for the Pfam [13] alignment database.

The purpose of the current study is to systematically assess the impact of the guide-tree on the quality on alignments of small numbers of protein sequences, where the accuracy can be measured using protein structure derived reference alignments. By confining ourselves to small numbers of sequences, we can systematically generate and test every possible guide-tree topology.

## Methods

We will construct guide-trees for different protein families with a small number of sequences and measure their respective alignment qualities, using several commonly used MSA programs on benchmark data derived from protein structure based alignments. The quality of the alignments is evaluated in terms of total column (TC) and sum-of-pairs (SP) score.

### Aligners

We consider MSA programs that are in common use, allow input of an external guide-tree and are fast. We used the following programs with the respective command-line arguments:

- ◦ Clustal Omega v1.1.0 [4] (default)
- ◦ MAFFT FFT-NS-i v7.029b [5]
–anysymbol –retree 1 –maxiterate 0 –unweight
- ◦ MAFFT L-INS-i v7.029b [5]
–anysymbol –retree 1 –maxiterate 0 –unweight
- ◦ MUSCLE v3.8.31 [6] -maxiters 1
- ◦ PAGAN v.0.47 [14] (default)

These aligners are based on related algorithmic approaches. Clustal Omega converts sequences and intermediate profiles into hidden Markov models (HMMs) and aligns these HMMs using HHalign [15]. By default it makes very fast guide-trees using the mBed algorithm [16] and does not use iteration, although both guide-tree and alignment can be iterated, on request. MUSCLE uses a standard profile-to-profile alignment method but is very highly optimised and makes extensive use of iteration to gradually improve the alignment and guide-tree. MAFFT L-INS-i uses consistency as introduced in [17] and as such, is only suitable for relatively small numbers of sequences. It also makes use of iteration. MAFFT FFT-NS-i uses Fast Fourier Transforms for very fast pairwise alignments and is the standard MAFFT program for fast high-throughput alignment of medium to large numbers of sequences. PAGAN uses a phylogeny-aware graph alignment algorithm and relies explicitly on having a phylogenetic tree as guide-tree. These have to be generated outside of PAGAN.

The main purpose of this study is to correlate alignment scores with particular tree topologies for the basic profile-profile alignment engine. Therefore we disable iteration, as this will modify the alignment order. By iteration we mean a process that attempts to improve the objective score by repeatedly adjusting an initial MSA. This process can entail a modification of the guide-tree or a re-alignment of individual sequences onto a preliminary alignment. Consequently the scores for MAFFT (FFT-NS-i/L-INS-i) and MUSCLE in this study are lower than in their respective default modes, where iteration is enabled. We do give these default scores in Additional file 1: Supplement S1.

To score an alignment we use the Total Column (TC) and Sum-of-Pairs (SP) scores, as implemented by qscore [6].

### Benchmark data

Benchmark data were extracted from the HOMSTRAD data base [18]. We selected single domain protein families with at least 5 sequences and multi-domain families with 8 or more sequences. If more than 8 sequences were available for a particular family then we reduced the number down to 8, picking the sequences randomly. If more than 12 sequences were available then we created an extra test family, possibly with re-sampling. If the number of protein structures was between 5 and 7, then we supplemented the sequences with homologous Pfam sequences [13]. In this case scoring of the alignment can only be done on the embedded HOMSTRAD reference alignment. We assembled 153 protein families, 75 were augmented with additional Pfam sequences and 15 were re-samples of larger HOMSTRAD families. Alignment lengths vary between 35 and 936, average sequence lengths vary between 28.5 and 780.8 and average pair-wise identities ranged between 14.76% and 77.55%. 15 families were comprised of multiple (up to 3) domains. A summary of reference family statistics can be found in Additional file 1: Supplement S2.

### Guide-trees

#### Distance based default guide-trees

Progressive alignment is a 2-stage process. The first stage is the guide-tree construction, the second stage comprises of the alignment of individual sequences and successively larger profiles, as specified by the guide-tree. In this study we will treat the second stage, that is the profile-profile aligner, as a ‘black box’ and focus on the first stage. In order to construct the guide-tree, many multiple sequence aligners construct a matrix of pair-wise distances. These distances can be k-tuple distances of unaligned sequences or full alignment distances. For small numbers of sequences, *N*, it is feasible to construct a full *N* × *N* distance matrix; if the number of sequences is large (usually *N* > 10,000), then time and memory may be conserved by calculating distances of the *N* sequences to only a small number of seeds, *n* ≪ *N* [16]. The distance matrix can then be converted into a guide-tree, using Neighbour-Joining or UPGMA algorithms. The version of PAGAN that we used in this analysis does not construct a default guide-tree. As mentioned earlier, we turn off all iterations, which would interfere with our guide-treeselection.

#### Guide-trees based on estimated phylogeny

We do not know the true phylogeny of the test sets we align but we do have high quality reference alignments. These were used to estimate the phylogeny using a range of methods. The best-fit empirical model of amino acid sequence evolution for each reference alignment was determined using ProtTest 3 [19]. Each model was determined using the Akaike Information Criterion (AIC) [20], corrected Akaike Information Criterion (AICc) [21], Bayesian Information Criterion (BIC) [22] and Decision Theory Criterion (DT) [23]. The most likely tree for each alignment was inferred using the maximum likelihood approach employed by RAxML [24]. In addition to the best-fit model of sequence evolution, the Generalised Time Reversible (GTR) model and GTR model where a fraction of amino acids is considered invariable (‘+I’) were used for each alignment. In all cases, GTR or GTR+I trees produced higher log likelihood scores than the best-fit model predicted using ProtTest 3, and so were considered to be the most likely tree available. For 78 families we had species information for all 8 sequences. Out of these we were able to root 43 trees by hand. Of these, 13 trees were mid-point rooted and another 9 trees had a Robinson-Foulds distance of 2 from the mid-point rooted tree. We also tried all 14 possible rootings for all 153 families and ranked the quality of the alignment, using the mid-point rooting. For Clustal Omega mid-point rooting was the best in 61/153 cases, for MUSCLE 45/153, for default MAFFT 55/153 and for MAFFT L-INS-i 54/153. We therefore used hand-rooted trees where they were available and thought it reasonable to use mid-point rooted trees, where no better tree could be obtained. Trees were (mid-point) rooted using PHYLIP’s retree command [25]. The list of estimated phylogenetic trees, henceforth called ML trees, can be found in Additional file 1: Supplement S3, where we also show that 133/153 trees are within 1*σ* of the imbalance expected under an equal rates Markov model [26].

#### Systematic guide-tree construction

The number of possible guide-trees grows with the number of sequences *N*. For a rooted tree the number of labeled guide-trees is *L*_*N*_ = (2*N*-3)!! [27]. In the present study we will analyse *L*_4_ = 15, *L*_5_ = 105, *L*_6_ = 945, *L*_7_ = 10, 395 and in particular *L*_8_ = 135, 135. No closed formula is known for the number of unlabeled guide-tree topologies *U*_*N*_, but in this study we use *U*_4_ = 2, *U*_5_ = 3, *U*_6_ = 6, *U*_7_ = 11 and *U*_8_ = 23 [28]. In general, there are *N*! ways to distribute *N* sequence labels amongst the leaves of a guide-tree; however, sequence alignment should be a symmetric process, so that every degree of symmetry decreases the number of topologically distinct labeled guide-trees by a factor of 2. For example, a perfectly balanced tree with 4 sequences ((1,2),(3,4)), has three degrees of symmetry, that is (1,2) ↔ (2,1), (3,4) ↔ (4,3) and ((1,2),(3,4)) ↔ ((3,4),(1,2)), so that there are *B*_4_ = 4!/2^3^ = 3 distinct balanced trees with 4 leaves. These are ((1,2),(3,4)), ((1,3),(2,4)) and ((1,4),(2,3)). A perfectly chained tree of 4 sequences (((1,2),3),4) has only one degree of symmetry, that is, (1,2) ↔ (2,1), so that there are *C*_4_ = 4!/2^1^ = 12 distinct chained trees of 4 sequences, given in Additional file 1: Supplement S4. There are no other topologically distinct unlabeled trees for 4 sequences. This example is consistent, as *B*_4_ + *C*_4_ = 3 + 12 = *L*_4_, which is the expected number of all labeled trees with 4 leaves.

We are particularly interested in the case of 8 sequences, as these trees can be perfectly balanced. This means that at every internal node there is an equal number of sequences subtended by both branches. Only trees with *N* leaves, where *N* is a power of 2, can be perfectly balanced. The next such tree has 16 sequences. For 16 sequences there are *U*_16_ = 10, 905 unlabeled trees and *L*_16_ ≈ 6.2 × 10^15^ labeled trees. A complete exploration for 16 sequences is outside the scope of this study. However, in Additional file 1: Supplement S5 we present results for 101 topologically distinct trees of 16 sequences, each labeled in 10,000 different ways. Perfectly imbalanced trees are trees where at every internal node (at least) one of the two branches subtends exactly one sequence. These trees are sometimes referred to as pectinate (comb-like) or linear; we call them chained. For *N* > 4 sequences there are, aside from perfectly balanced and perfectly chained trees, trees of an intermediate degree of balance. Several measures to quantify this degree are in use, for example, Sackin’s index [29], the index described by Colless [30], the inverse-maximum index, as described by Sokal [31] and Shannon entropy. However, apart from the perfectly balanced and chained trees, none of these indices give exactly the same ranking of trees (for 8 or more sequences), so that the ordering of trees according to their degree of balance is somewhat fuzzy and depends on the specific aspect of the property measured by the respective index. In Additional file 1: Supplement S6 we show all unlabeled guide-trees for 4, 5, 6, 7 and 8 sequences and quote their respective measures of im/balance.

### Different clustering schemes

Apart from the aligners’ default and the ML trees we tried various other clustering schemes, as outlined in [32]. We consider Single Linkage (SL), merging clusters for which the minimum distance between their elements is the least one; Complete Linkage (CL), merging clusters for which the maximum distance between their elements is the least one; Mean Linkage (MeanL), merging clusters for which the Euclidean distance between their centroids or means is the least one; Ward’s Criterion, merging clusters for which the increase in variance for the resulting group is the least one. In addition, we considered UPGMA and Neighbour Joining trees, as produced by Clustal W2 [33].

### Populating chained guide-trees

It has been shown [10] that randomly populated chained guide-trees *on average* produce good alignments. However, any particular randomly populated chained guide-tree might in fact produce a bad alignment. One would like to select an ordering with the best possible outcome. In order to determine such an ordering scheme we will arrange the sequences according to their length, hydrophobic moment (HM), isoelectric point (IP) and sequence similarity. For the HM and IP we consider absolute values and values normalised by the sequence length. HM and IP are calculated according to [34]. For all criteria we sort in ascending and descending order. Sequences cannot always be uniquely sorted according to just one sort key, there may be ties. We also explore secondary sort keys.

### Benchmarking

For each protein family, we allowed each aligner to construct a default guide-tree. Using this default guide-tree the aligners construct an initial alignment, without iteration. We call this alignment the default-tree alignment as it uses the default guide-tree, despite not using default command-line flags. The version of PAGAN that we used does not construct a default guide-tree. In a next step we use the ML tree as the guide-tree, again without iteration. We then used all possible guide-trees to align the sequences. The alignments were scored using qscore. We collected the TC score, which is defined as the fraction of correctly aligned core columns of residues of all core columns in the alignment. Core columns were determined where the structural superposition for every reference sequence agreed. We accepted helix, sheet and coil states, since the Euclidean distance between each pair of alpha carbons within the column was within a threshold of 0.3 nm [35]. We rejected the JOY criterion [18], where only 70% of sequences have to agree, as this produced too many columns, which by visual inspection could not be deemed reliably aligned. This reduces the ranges of lengths of the alignments from [35:936] to [6:526], the largest percent reduction was down to 12.2% (74 →9), while the largest retention was 88.6% (323 →286). The behaviour of four example families can be found in Additional file 1: Supplement S7. The tree/s yielding the highest TC score is/are then easily identified by sorting the 135,135 TC scores for the different trees. We count how many trees produce the same, highest TC score.

## Results

### Estimated phylogenetic trees

**Figure 1:**
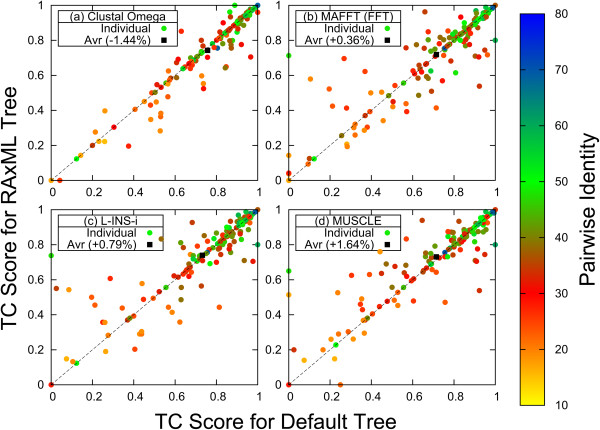
**Comparison of TC Scores for default and ML tree.** TC scores for default tree along x-axis, for phylogenetic tree along y-axis for **(a)** Clustal Omega, **(b)** MAFFT FFT-NS-i, **(c)** MAFFT L-INS-i, **(d)** MUSCLE. Colour dots are used for individual protein families: Blue and green for high percentage identity reference alignments, yellow and red for low identity. Black box is used for average TC score. Below the dotted line the default tree is better than the ML tree, above the ML tree is better than the default tree.

Figure 1 compares the TC scores for the 153 test families with 8 sequences, using default guide-trees versus ML trees, for the various aligners. Along the x-axis are the TC scores when using default trees; along the y-axis when using ML trees. The coloured dots represent results for each of the 153 protein families, the colour encodes the average percentage identity of the reference alignment. Unsurprisingly one sees that in general, high identities (blue and green) are associated with high scores (top-right corner) while low identities (yellow and red) are associated with low scores (bottom-left corner). A dot below the bisectrix (dotted line) means that the default tree gives a better score than the ML tree. A dot above the bisectrix means that the ML tree produces a better score than the default tree. The black squares represent the average of all 153 alignments. We notice that for Clustal Omega the ML trees produce, on average, worse results than the default trees (-1.44%), for MAFFT FFT-NS-i and L-INS-i the results are slightly better (+0.36% and +0.79%, respectively). ML trees produce a better average score for MUSCLE (+1.64%). For Clustal Omega the individual data points are tightly arranged along the bisectrix. For MAFFT FFT-NS-i, L-INS-i and MUSCLE the ML trees appear to give an improvement for difficult alignments and a deterioration for easy alignments. Results using the SP score can be found in Additional file 1: Supplement S8. As there is no default tree for PAGAN there is no corresponding PAGAN panel in Figure 1.

### Best possible trees

**Figure 2:**
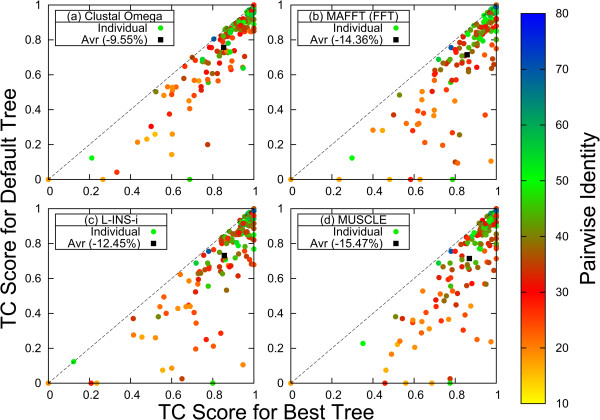
**Comparison of TC Scores for default and best tree.** TC scores for best tree along x-axis, for default tree along y-axis for **(a)** Clustal Omega, **(b)** MAFFT FFT-NS-i, **(c)** MAFFT L-INS-i, **(d)** MUSCLE. Colour dots are used for individual protein families: Blue and green for high percentage identity reference alignments, yellow and red for low identity. Black box is used for average TC score. Here all points must be below bisectrix as no tree can be better than the best tree.

Figure 2 compares the default TC scores for the 153 test families with 8 sequences, with the best TC scores found from scores for the 135,135 possible guide-trees. Clearly, no points can lie above the bisectrix. Points on the bisectrix represent the best possible outcome. We notice that the default trees for all aligners fall way short of the best possible outcome, that is -9.55% for Clustal Omega, -14.36% for MAFFT FFT-NT-i, -12.45% for L-INS-i and -15.47% for MUSCLE. As there is no default tree for PAGAN there is no corresponding panel in Figure 2.

**Figure 3:**
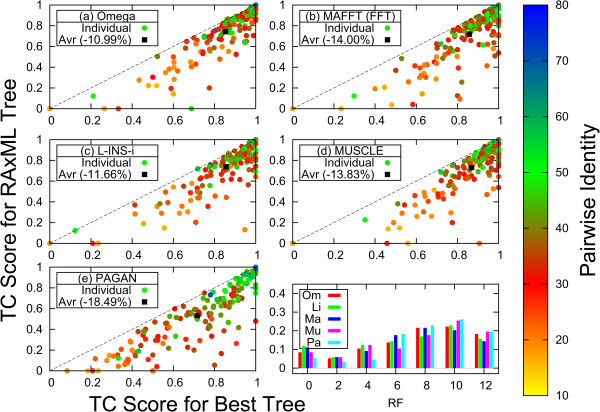
**Comparison of results for best possible and ML tree.** TC scores for the best tree along x-axis, for ML tree along y-axis for **(a)** Clustal Omega, **(b)** MAFFT FFT-NS-i, **(c)** MAFFT L-INS-i, **(d)** MUSCLE, **(e)** PAGAN. Colour dots results are used for individual families, black squares averages of families. Bottom right-hand panel distribution of Robinson-Foulds distances between best and ML tree. Frequencies for Clustal Omega (Om) shown in red, MAFFT L-INS-i (Li) in green, MAFFT FFT-NS-i (Ma) in dark blue, MUSCLE (Mu) in magenta and PAGAN (Pa) in light blue.

Figure 3 compares the best possible results for the 153 test families with 8 sequences, with the results for the alignments obtained with the ML trees; this figure does show results for PAGAN in panel (e). The average TC scores for the ML tree fall way short of the best possible TC scores; this is consistent with Figure 2.

In the bottom right-hand panel of Figure 3 we show the proportion of times the ML guide-tree has a certain distance from the guide-tree giving the best possible TC score, as measured by the Robinson-Foulds (RF) metric for rooted trees [36]. Trees that are isomorphic and label preserving have an RF distance of 0, the maximum RF distance for two rooted trees with 8 sequences is 12. If for any family more than one tree produced the same highest score we registered the tree with the lowest RF distance. The bottom right-hand panel of Figure 3 shows that in less than 12% (5.2%–11.8%) of the families the best possible tree is isomorphic and label preserving (RF = 0) wrt the ML tree. On the other hand for roughly a sixth (14.4%–19.6%) of families the ML tree is as far away from the best tree as possible (RF = 12) under the RF metric. The average RF distances for the different aligners are 7.49 (Clustal Omega), 7.01 (L-INS-i), 7.02 (MAFFT FFT-NS-i), 7.56 (MUSCLE), 8.14 (PAGAN) – all more than half the maximum distance.

### Different clustering schemes

Table 1 shows the performance of various clustering schemes for the different aligners. The best scheme for PAGAN in this study is the mean or centroid linkage, which merges clusters for which the Euclidean distance between their centroids or means is the least one. For all other aligners Single Linkage appears to be the best scheme, that is, better than their respective default guide-tree, whose entries in the first row correspond to the average values in Figures 1 and 2.

**Table 1:** **TC scores for different aligners and clustering schemes**

	Clustal-Omega	MUSCLE	MAFFT	L-INS-i	PAGAN
Default	0.757	0.722	0.718	**0.754**	NA
RAxML	0.743	0.731	0.718	0.740	0.532
UPGMA	0.753	0.738	0.734	0.749	0.535
NJ	0.744	0.725	0.711	0.735	0.527
Single	**0.759**	**0.744**	**0.736**	**0.754**	0.535
Complete	0.742	0.732	0.721	0.735	0.526
Mean	0.750	0.737	0.730	0.742	**0.541**
Ward	0.711	0.700	0.686	0.709	0.498

Default guide-tree for respective aligner, ‘RAxML’ score for ML tree produced from *true* alignment, UPGMA/NJ trees produced by ClustalW2, single/complete/mean linkage clustering schemes, ‘Ward’ variance minimising scheme. Best scheme for each aligner in bold.

### Populating chained trees

Table 2 gives TC scores for perfectly chained trees, where the leaves were populated with sequences in a certain order. For the current benchmark data of alignable and homologous sequences it appeared to be generally more beneficial to arrange sequences in ascending order of length, that is, to align short sequences before long ones. For fragmented, non-overlapping sequences this would presumably not be a good strategy. Data for the hydrophobic moment are not conclusive, because it appears to be beneficial to arrange the sequences in order of descending *absolute* HM, while the best ordering for the HM divided by the respective sequence length is ascending. For the isoelectric point the best ordering appears to be ascending. The strongest predictor for a good ordering, however, appears to be sequence identity. We consider the highest and/or lowest identity of a sequence to any other sequence. Here it is clearly best to first align similar sequences. This ordering gave the best scores for MUSCLE, MAFFT FFT-NS-i and PAGAN, that is, it was better than their respective default guide-tree. For Clustal Omega and MAFFT L-INS-i this ordering was second best, only slightly worse than the default tree.

**Table 2:** **TC scores for chained guide-trees populated according to certain criteria**

Aligner:	Clustal Omega	MUSCLE	MAFFT	L-INS-i	PAGAN
len/a	0.731	0.711	0.697	0.723	0.453
len/d	0.700	0.693	0.671	0.684	0.447
HM/a	0.705	0.704	0.680	0.696	0.451
HM/d	0.717	0.697	0.693	0.710	0.454
HML/a	0.709	0.718	0.695	0.706	0.458
HML/d	0.706	0.692	0.678	0.695	0.453
IP/a	0.731	0.701	0.692	0.717	0.466
IP/d	0.698	0.699	0.671	0.693	0.459
IPL/a	0.717	0.701	0.691	0.707	0.449
IPL/d	0.705	0.704	0.683	0.705	0.454
hi/a	0.685	0.685	0.649	0.674	0.423
hi/d	0.745	**0.731**	**0.724**	0.742	**0.483**
lo/a	0.654	0.641	0.598	0.614	0.397
lo/d	0.730	0.722	0.717	0.729	0.461
def	**0.757**	0.722	0.718	**0.754**	NA
RAxML	0.743	0.731	0.718	0.740	0.532

‘len/a’ length ascending, ‘len/d’ length descending, ‘HM/a’ hydrophobic moment ascending, ‘HM/d’ HM descending, ‘HML/a’ hydrophobic moment divided by sequence length ascending, ‘HML/d’ HML descending, ‘IP/a’ isoelectric point ascending, ‘IP/d’ IP descending, ‘IPL/a’ isoelectric point divided by sequence length ascending, ‘IPL/d’ IPL descending, ‘hi/a’ highest sequence similarity ascending, ‘hi/d’ highest similarity descending, ‘lo/a’ lowest sequence similarity ascending, ‘lo/d’ lowest similarity descending. ‘def’ is the default guide tree (non for PAGAN), RAxML is the ML tree estimated from the *true* reference alignment. The best score for every aligner is highlighted in bold (unless RAxML).

### Tree branch lengths

**Figure 4:**
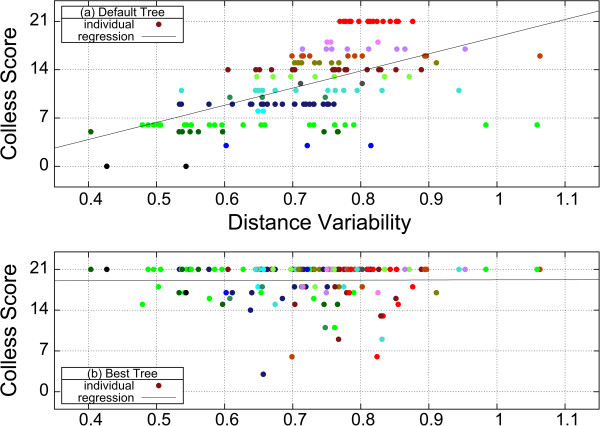
**Effect of branch length variability on default and optimum tree shape.** Panel **(a)** correlates variability of distances with the degree of imbalance for the default tree. Families are represented with dots, the colour encoding the Colless score. Panel **(b)** correlates variability of distances with the degree of imbalance for an optimum tree. Families represented by the same colour as in panel **(a)**.

The analysis so far has not taken absolute or relative tree branch lengths into consideration. The question therefore arises if the distribution of tree branch lengths has an effect. For each family we determined the variability in the distribution of branch lengths. In the top panel (a) of Figure 4 the Colless measure of imbalance of the default guide-tree was plotted against this variability. Data points with the same Colless score are rendered in the same colour. On average (straight line) one observes that low variability gives rise to more balanced trees (low Colless numbers), while higher variability causes the trees to be more chained (high Colless numbers), that is, in panel (a) the black line is rising. Next, the optimum trees were identified. In the bottom panel (b) the Colless measure of imbalance for the optimum trees is plotted against the variability of the branch lengths. Here no correlation between variability and topology is discernible, that is, the black line is flat. It is possible to match up the differently coloured dots, spanning the entire spectrum of variability: most are shifted towards a high degree of imbalance, many of them are perfectly chained. We conclude that the variability of branch lengths does not affect whether the optimum tree is balanced or chained. Some examples with extreme topology and/or branch length distribution are shown in Additional file 1: Supplement S11.

### Optimum guide-tree discrimination

**Figure 5:**
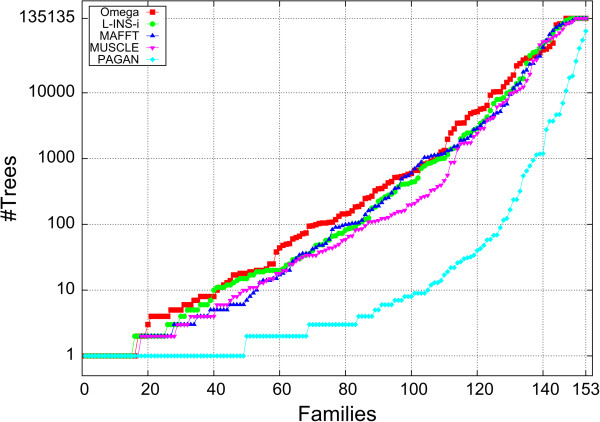
**Number of trees that produce optimum TC score.** Along the x-axis number of families with no more trees producing optimum TC score than indicated along y-axis. Clustal Omega shown with red boxes, MAFFT L-INS-i with green bullets, MAFFT FFT-NS-i with dark blue triangles, MUSCLE with inverted magenta triangles, PAGAN with pale blue diamonds.

When evaluating all possible 135,135 guide-trees for 8 sequences we determined the best TC score attained for each aligner and counted how many different trees gave rise to this score. For some families only one unique guide-tree produced the optimum alignment. For some families multiple trees produced the optimum alignment and for others families the guide-tree was completely irrelevant and all 135,135 trees produced the same alignment. This behaviour is depicted in Figure 5.

Figure 5 shows how many families have at most a certain number of guide-trees which produce the highest TC score. For example, for Clustal Omega there are 16 protein families whose highest TC score is produced by exactly one guide-tree. For MAFFT FFT-NS-i/L-INS-i, MUSCLE and PAGAN these numbers are 17, 15, 17 and 49, respectively. On the other hand, there are 6, 3, 4, 3 families where all 135,135 guide-trees produce the same TC score for Clustal Omega, MAFFT FFT-NS-i, L-INS-i and MUSCLE; there is no such family for PAGAN, where the most ‘promiscuous’ family has 87,063 ‘optimum’ guide-trees. It should be noted that the unique and promiscuous families are not necessarily the same for each aligner. In fact there are only five families, where each aligner only identifies exactly one optimum tree, and only two families where, apart from PAGAN, all aligners find that all trees are equivalent.

### Average TC score for different topologies

**Figure 6:**
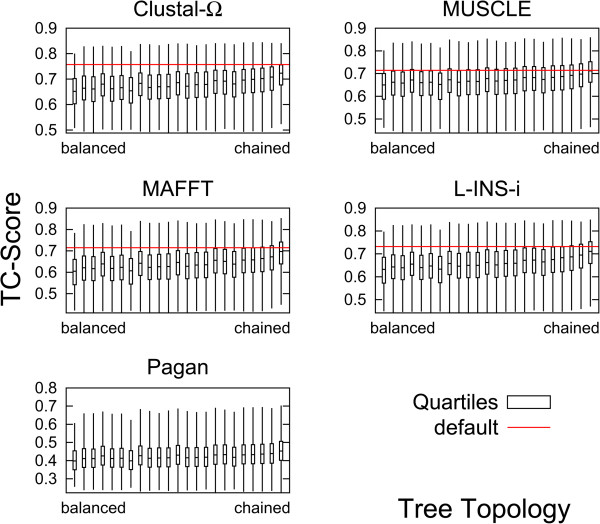
**Quartiles of TC scores for different tree topologies.** Tree topology along the x-axis, left-most box for perfectly balanced tree, right-most box for perfectly chained tree, intermediate topologies as specified in Additional file 1: Supplement S6. Whiskers represent top/bottom 25% scores, band represents median score. Boxes are averages over all 153 protein families. Red horizontal line shows average default score.

Figure 6 shows the average TC score for different guide-tree topologies for 8 sequences. The whiskers show the top and bottom 25%: the band inside the box shows the median. TC scores are averaged over all 153 protein families. The horizontal red line shows the average TC score for the default guide-tree. The 23 guide-tree topologies are ordered from left to right with increasing degree of imbalance; labeling and the concept of imbalance is explained in Table S2 of Additional file 1: Supplement S6. The first box is for the perfectly balanced guide-tree, the last box for the perfectly chained one.

We first observe a distinct increase in the TC scores with increasing imbalance. This means that a randomly labeled chained tree is on average better than a randomly labeled balanced tree. This is true for all aligners considered in this study. On the other hand, it is not true to say that all balanced trees produce bad alignments, as the top whisker of the balanced box well overlaps with the top whisker of the chained box. Secondly, we observe that the default score is always above the median score for any of the guide-trees. This means that the default guide-tree for 8 sequences on average is better than a randomly populated guide-tree, whether it be chained or balanced. This is particularly true for L-INS-i and Clustal Omega, however for MAFFT FFT-NS-i and especially MUSCLE the default guide-tree is on average only marginally better than a randomly labeled chained guide-tree. This is also true for 16 or fewer sequences, as shown in Additional file 1: Supplement S9. In Additional file 1: Supplement S10 we show how often certain tree topologies produced the best and the worst results.

## Discussion

We found that for Clustal Omega and MAFFT L-INS-i TC scores for the default tree and the ML tree are tightly correlated. This may be in part due to the underlying profile-profile alignment strategy. Clustal Omega uses HMMs and L-INS-i uses consistency; both appear to confer a certain degree of ‘robustness’ wrt the choice of guide-tree. For MAFFT FFT-NS-i and particularly for MUSCLE we found that phylogeny based guide-trees produce a small improvement over default trees for difficult alignments and a deterioration for easy alignments. Here the underlying alignment engine is more susceptible to a sub-optimal guide-tree, and the quality of the alignment depends more on the choice of a good guide-tree. In their respective default modes MUSCLE and MAFFT FFT-NS-i compensate for this by iteration. On average we found that ML guide-trees are not better than default distance based guide-trees when performing a progressive alignment. This has long been suspected [37]. The argument there is that sequences with the highest identity can be aligned most accurately. However, if phylogenetic rates vary considerably among lineages, then the evolutionary neighbour may not be the nearest neighbour wrt identity. We see evidence for this conjecture by comparing TC scores for both strategies as well as analysing the Robinson-Foulds distances.

While the differences in TC scores are small between ML and default guide-trees, there is a vast potential when compared with results for the best possible trees. It would be worthwhile to try to devise better guide-tree construction schemes, especially since contributions from the guide-tree to the alignment accuracy appear to decouple from contributions from the profile-profile alignment stage while the overall accuracy is bound to decrease for larger numbers of sequences [38].

A structure based evaluation is only one possible angle on benchmarking as it does not primarily test gap placement due to insertion/deletion events [39]. We could confirm that PAGAN is by far the most phylogeny aware aligner amongst the ones considered in this study, despite being evaluated on a non-phylogeny based benchmark strategy. The other aligners displayed a similar degree of awareness in discriminating between good and bad guide-trees (evaluated on a protein structure based reference alignment), with MUSCLE being slightly more sensitive than the other three.

When grouping alignment scores according to guide-tree topology we found that chained guide-trees, on average, produce better results than balanced ones. This seems to run counter the established wisdom of trying to balance guide-trees but can be understood when realising that chained trees have fewer sequence pairs that cross the root and the mean pair-wise distance therefore being less than for a balanced tree [40]. For the small numbers of sequences we analysed, we could not confirm that a randomly labeled chained guide-tree is better than the default guide-tree. However, as the number of sequences is increased from 4 to 8 and then to 16 this difference appears to decrease, and we suspect that beyond a certain number of sequences, randomly labeled chained guide-trees will be better than distance based default guide-trees, see Additional file 1: Supplement S12. This is consistent with findings in [10] who observed that for the small numbers of sequences in BAliBASE 3.0 [41] randomly labeled chained trees were sometimes as good as default trees, while for more than 1,000 sequences randomly labeled chained trees were clearly better. This suggests that the greatest (and easiest) improvements of guide-tree construction may come from finding an optimum non-random labeling strategy for chained trees.

## Conclusions

Alignment methods that use Consistency or hidden Markov models to make alignments are less susceptible to sub-optimal guide-trees than simpler methods, that basically use conventional sequence alignment between profiles. The latter appear to be affected positively by evolutionary based guide-trees for difficult alignments and negatively for easy alignments. One phylogeny aware alignment program can strongly discriminate between good and bad guide-trees. The results for randomly chained guide-trees improve with the number of sequences.

## Availability of supporting data

Benchmark sequences, tree topologies, utility programs and driver scripts are available as http://www.bioinf.ucd.ie/download/BMC-2014-treeExploration.tar.gz.

## Electronic supplementary material

Additional file 1: **Supplemental material.** (PDF 821 KB)
