# Supplementary material for: Systematic exploration of guide-tree topology effects for small protein alignments
Source: BMC Bioinformatics. 2014 Oct 4;15(1):338. doi: 10.1186/1471-2105-15-338 (PMC4287568; doi:10.1186/1471-2105-15-338)
Supplement: Supplementary file 1 — Additional file 1: Supplemental material. (PDF 821 KB) [file 12859_2014_6641_MOESM1_ESM.pdf]

RESEARCH

# Systematic Exploration of Guide-Tree Topology Effects for Small Protein Alignments

Fabian Sievers*, Graham M Hughes and Desmond G Higgins

*Correspondence:
fabian.sievers@ucd.ie
University College Dublin, Conway Institute, Dublin, Ireland
Full list of author information is available at the end of the article

## Abstract

**Background:** Guide-trees are used as part of an essential heuristic to enable the calculation of multiple sequence alignments. They have been the focus of much method development but there has been little effort at determining systematically, which guide-trees, if any, give the best alignments. Some guide-tree construction schemes are based on pair-wise distances amongst unaligned sequences. Others try to emulate an underlying evolutionary tree and involve various iteration methods.

**Results:** We explore all possible guide-trees for a set of protein alignments of up to eight sequences. We find that pairwise distance based default guide-trees sometimes outperform evolutionary guide-trees, as measured by structure derived reference alignments. However, default guide-trees fall way short of the optimum attainable scores. On average chained guide-trees perform better than balanced ones but are not better than default guide-trees for small alignments.

**Conclusions:** Alignment methods that use Consistency or hidden Markov models to make alignments are less susceptible to sub-optimal guide-trees than simpler methods, that basically use conventional sequence alignment between profiles. The latter appear to be affected positively by evolutionary based guide-trees for difficult alignments and negatively for easy alignments. One phylogeny aware alignment program can strongly discriminate between good and bad guide-trees. The results for randomly chained guide-trees improve with the number of sequences.

**Keywords:** Multiple Sequence Alignment; Guide-Tree Topology; Alignment Accuracy; Benchmarking

## Supplemental Material

(S1) Iterated vs Non-Iterated Alignments

When evaluating the effect of the guide-trees we turned off all iterations. This change only applied to MUSCLE and MAFFT (FFT-NS-i/L-INS-i). We did this not because we deliberately wanted to reduce the accuracy of these aligners but to avoid reconstruction of the guide-trees. These aligners achieve a good improvement in the alignment score through the iteration procedure. These improvements were on average +1.48% for MAFFT FFT-NS-i, +1.25% for L-INS-i and +3.19% for MUSCLE. Supplemental Figure (1S) shows the effect of turning iteration on and off.

(S2) Simple Statistics on Reference Alignments

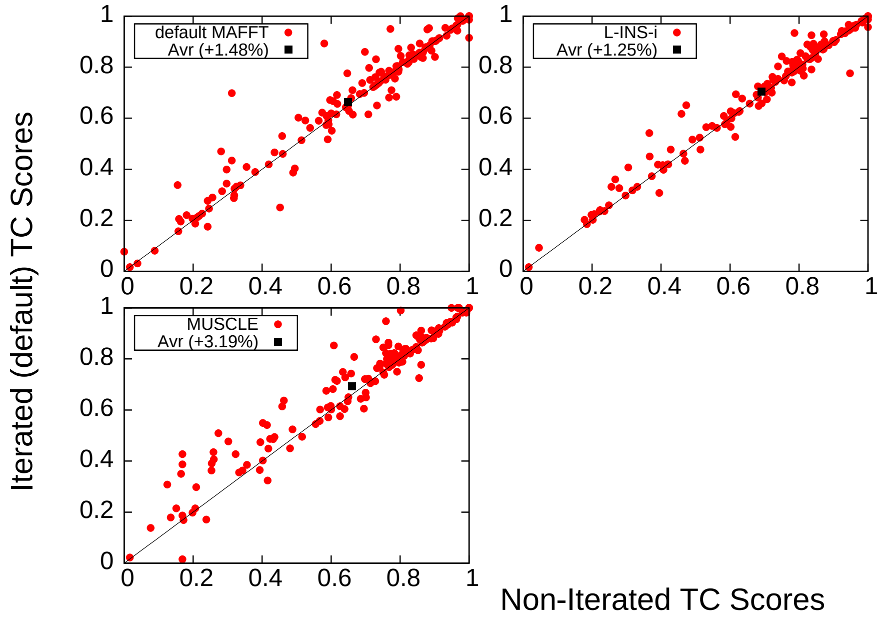


**Figure 1 Effect of Iteration for MAFFT and MUSCLE** Not-iterated TC score –as used in this study– along the x-axis. Iterated (default) TC score along the y-axis. Red dots for individual protein families, black box for average of 153 families. Default (iteration) better than non-iteration above bisectrix. Results for MAFFT FFT-NS-i/L-INS-i and MUSCLE

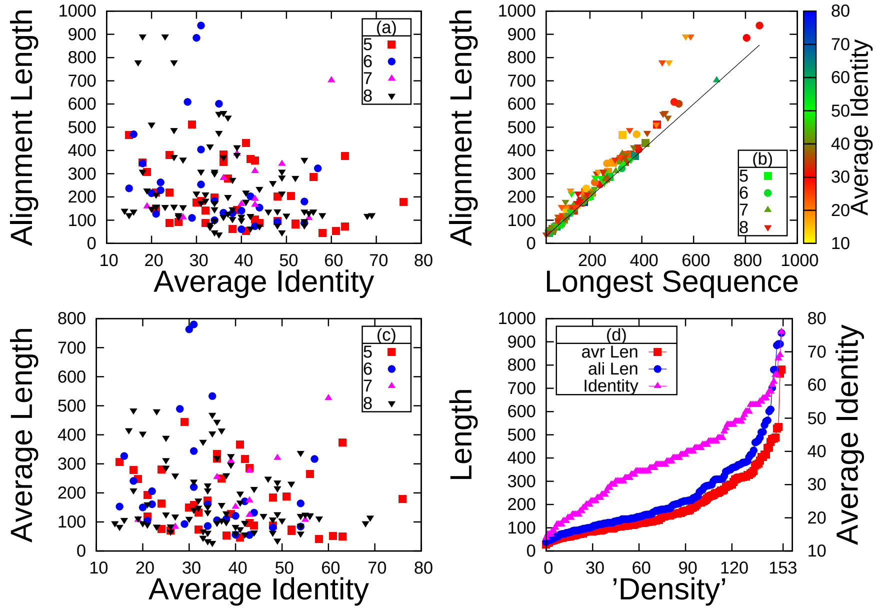


**Figure 2 Simple Statistics on Reference Alignments** (a) Alignment length vs average identity, alignments comprised of 5 sequences with red squares, of 6 with blue bullets, of 7 with magenta triangles. Alignments of the full complement of 8 sequences with black upside-down triangles. (b) Alignment length vs the length of the longest sequence in the alignment. Shape of the symbols same as in panel (a), however, colour encodes average pairwise identity (high ID blue/green, low ID red/yellow). (c) Average sequences length vs average identity, key same as panel (a). (d) Number of families (along x-axis) where average identity (magenta triangles), alignment length (blue bullets) and average sequence length (red boxes) is less than a certain value (along y-axes)

Figure (2S) shows some simple statistics –as produced by Sean Eddy's program `alistat`– for the 153 reference alignments for alignments of 8 sequences used in this study. All test sets in the main part of this study are comprised of 8 sequences; however, the reference alignments sometimes are comprised of fewer than 8 sequences. If only 5, 6 or 7 HOMSTRAD reference sequences were available, then we used homologous Pfam sequences to make up the full complement of 8. Alignment quality, however, can only be assessed based on the HOMSTRAD reference sequences. Figure (2S) shows statistics for these references. No family contains fewer than 5 reference sequences.

37 reference families are comprised of 5 sequences (supplemented with 3 Pfam sequences), rendered in panels a-c of Figure S2 as square boxes. 27 reference families are composed of 6 HOMSTRAD sequences (supplemented with 2 Pfam sequences) rendered as bullets. 11 families consist of 7 HOMSTRAD reference sequences (supplemented with 1 Pfam sequence), rendered as triangles. 78 families have the full complement of 8 HOMSTRAD reference sequences. Out of these, 12 families consisted of two protein domains, 3 families of three domains; all other families consisted of one domain. The average pairwise identity of the reference alignments ranges from 14.76% to 77.55%. These are plotted along the x-axes of panels a and c, along the rhs-y-axis of panel d and are rendered as colour in panel b. The average sequence lengths range from 28.5 to 780.8 and are plotted along the y-axes of panels c and d. Alignment lengths vary between 35 and 936, and are plotted along the y-axes of panels a,b,d. The length of the longest sequence in each family is the smallest possible length of the alignment. This number varies between 31 and 854, and is plotted along the x-axis of panel b.

### (S3) Expected Imbalance under Equal Rates Markov Model

The index of imbalance, according to Colless [30] can be written as

$$I^{(C)} = \sum_{i=1}^{k} \Delta_i$$

where $i$ runs over the number of interior nodes –in MSA all of degree 3– and where $\Delta_i$ is the absolute difference in number of terminal nodes subtended by the two branches of bifurcation i. This index can vary for a tree with $N$ leaves between 0 (totally balanced) and $(N-1)(N-2)/2$ (totally chained). It was shown [26] that under an equal rates Markov model the expected value for the Colless index and its variance can be calculated recursively as

$$\begin{aligned}
E\left[I_N^{(C)}\right] &= \frac{1}{N-1}\sum_{i=1}^{N-1}\left(2E\left[I_i^{(C)}\right] + |N-2i|\right)\\
V\left[I_N^{(C)}\right] &= E\left[\left(I_N^{(C)}\right)^2\right] - E\left[I_N^{(C)}\right]^2\\
E\left[\left(I_N^{(C)}\right)^2\right] &= \frac{1}{N-1}\sum_{i=1}^{N-1} 2E\left[\left(I_i^{(C)}\right)^2\right] + 2E\left[I_{N-1}^{(C)}\right]E\left[I_i^{(C)}\right]\\
&\quad +4|N-2i|E\left[I_i^{(C)}\right] + |N-2i|^2
\end{aligned}$$

(1)

For 8 leaves the expected Colless imbalance index is 8.66667, the standard variation 5.818, while we measure 8.86275 for the estimated phylogenies of the reference alignments. 133 of the 153 families fall within $1\sigma$ around the expected mean.

## (S4) List of Labeled Trees with Four Leaves

Balanced:

((1,2),(3,4)) — ((1,3),(2,4)) — ((1,4),(2,3))

Chained:

(((1,2),3),4) — (((1,2),4),3) — (((1,3),2),4) — (((1,3),4),2) — (((1,4),2),3) — (((1,4),3),2) (((2,3),1),4) — (((2,3),4),1) — (((2,4),1),3) — (((2,4),3),1) — (((3,4),1),2) — (((3,4),2),1)

## (S5) Tree Exploration for 16 Leaves

For 16 sequences there are 10,905 topologically distinct unlabeled guide-trees and 6,190,283,353,629,375 distinct labeled trees. Evaluation (aligning and scoring) for so many guide-trees would take too long. We therefore decided to reduce the above numbers. On the one hand we wanted to sample trees of as many degrees of im/balance as possible. On the other hand we wanted to reshuffle the leaves for each topologically distinct tree as often as possible, while keeping the product of the number of trees and the number of re-samples feasible. The range of possible Colless indices for $N = 16$ is $[0 : 105]$, however, when constructing unlabeled trees we did only encounter 101 different values (there were no trees with 1,2,4,103,104). We randomly picked one tree topology from all the topologies with a certain Colless index. We felt that we could afford to evaluate around one million trees for each family and settled on 10,000 reshuffles per tree. There are $16!/2^{15} = 638,512,875$ distinctly labeled balanced trees and $16!/2 \approx 10^{13}$ distinctly labeled chained trees. Therefore our sampling is only a small proportion of all the labeled and unlabeled trees.

## (S6) Measures of Im/Balance for Small Trees

Tables S1 and S2 show measures of im/balance for trees with 2 to 8 leaves. The trees are rendered with nested parentheses, similar to Newick format. Next we quote the 'Depth', which is the maximum distance (in terms of number of internal nodes) from the root. 'S' is the Sackin measure [29] and 'C' is the Colless measure as defined in supplement S3 and originally in [30]. 'Inv-Max' is the imbalance measure defined in [31]. 'Entropy' refers to the Shannon Entropy. 'Dia' is the tree diameter. Table S2 precedes the 'Tree' column by the identifying index 'ID'; data in Figure (6) are arranged by this index. The last column in Table S2 quotes the number of distinct ways the tree can be labeled. Figure (3S) gives a graphic representation of the trees with eight leaves, as used in this study.

## (7S) Core vs Non-Core Scoring

Benchmarks like BAliBASE 3.0 use 'core columns' for scoring the alignments. The idea behind this concept is that residues outside of these core regions may not be reliably aligned in the reference alignment. It is therefore not appropriate to use these

**Table 1** Im/balance measures for trees with 2-7 leaves

| Tree | Depth | S | C | Inv-Max | Entropy | Dia |
|---|---|---|---|---|---|---|
| (1,2) | 1 | 2 | 0 | 0.000000 | 0.301030 | 3 |
| (1,(2,3)) | 2 | 5 | 1 | 1.000000 | 0.451545 | 4 |
| (1,(2,(3,4))) | 3 | 9 | 3 | 1.500000 | 0.526802 | 5 |
| ((1,2),(3,4)) | 2 | 8 | 0 | 2.000000 | 0.602060 | 5 |
| (1,(2,(3,(4,5)))) | 4 | 14 | 6 | 1.833333 | 0.564431 | 6 |
| (1,((2,3),(4,5))) | 3 | 13 | 3 | 2.500000 | 0.602060 | 5 |
| ((1,2),(3,(4,5))) | 3 | 12 | 2 | 2.500000 | 0.677317 | 6 |
| (1,(2,(3,(4,(5,6))))) | 5 | 20 | 10 | 2.083333 | 0.583246 | 7 |
| (1,(2,((3,4),(5,6)))) | 4 | 19 | 7 | 2.833333 | 0.602060 | 6 |
| (1,((2,3),(4,(5,6)))) | 4 | 18 | 6 | 2.833333 | 0.639689 | 6 |
| ((1,2),(3,(4,(5,6)))) | 4 | 17 | 5 | 2.833333 | 0.714946 | 7 |
| ((1,2),((3,4),(5,6))) | 3 | 16 | 2 | 3.500000 | 0.752575 | 6 |
| ((1,(2,3)),(4,(5,6))) | 3 | 16 | 2 | 3.000000 | 0.752575 | 7 |
| (1,(2,(3,(4,(5,(6,7)))))) | 6 | 27 | 15 | 2.283333 | 0.592653 | 8 |
| (1,(2,(3,((4,5),(6,7))))) | 5 | 26 | 12 | 3.083333 | 0.602060 | 7 |
| (1,(2,((3,4),(5,(6,7))))) | 5 | 25 | 11 | 3.083333 | 0.620874 | 7 |
| (1,((2,3),(4,(5,(6,7))))) | 5 | 24 | 10 | 3.083333 | 0.658503 | 7 |
| ((1,2),(3,(4,(5,(6,7))))) | 5 | 23 | 9 | 3.083333 | 0.733761 | 8 |
| (1,((2,3),((4,5),(6,7)))) | 4 | 23 | 7 | 3.833333 | 0.677317 | 6 |
| (1,((2,(3,4)),(5,(6,7)))) | 4 | 23 | 7 | 3.333333 | 0.677317 | 7 |
| ((1,2),(3,((4,5),(6,7)))) | 4 | 22 | 6 | 3.833333 | 0.752575 | 7 |
| ((1,2),((3,4),(5,(6,7)))) | 4 | 21 | 5 | 3.833333 | 0.790204 | 7 |
| ((1,(2,3)),(4,(5,(6,7)))) | 4 | 21 | 5 | 3.333333 | 0.790204 | 8 |
| ((1,(2,3)),((4,5),(6,7))) | 3 | 20 | 2 | 4.000000 | 0.827832 | 7 |

**Table 2** Im/balance measures for trees with 8 leaves

| ID | Tree | Depth | S | C | Inv-Max | Entropy | Dia | # |
|---|---|---|---|---|---|---|---|---|
| 1 | (((1,2),(3,4)),((5,6),(7,8))) | 3 | 24 | 0 | 5.000000 | 0.903090 | 7 | 315 |
| 2 | ((1,(2,(3,4))),((5,6),(7,8))) | 4 | 25 | 3 | 4.333333 | 0.865461 | 8 | 2520 |
| 3 | ((1,(2,3)),((4,5),(6,(7,8)))) | 4 | 25 | 5 | 4.333333 | 0.865461 | 8 | 5040 |
| 4 | ((1,(2,(3,4))),(5,(6,(7,8)))) | 4 | 26 | 6 | 3.666667 | 0.827832 | 9 | 5040 |
| 5 | ((1,2),((3,(4,5)),(6,(7,8)))) | 4 | 26 | 6 | 4.333333 | 0.827832 | 7 | 2520 |
| 6 | ((1,(2,3)),(4,((5,6),(7,8)))) | 4 | 26 | 6 | 4.333333 | 0.827832 | 8 | 2520 |
| 7 | ((1,2),((3,4),((5,6),(7,8)))) | 4 | 26 | 6 | 4.833333 | 0.827832 | 7 | 1260 |
| 8 | ((1,(2,3)),(4,(5,(6,(7,8))))) | 5 | 27 | 9 | 3.583333 | 0.809018 | 9 | 10080 |
| 9 | ((1,2),((3,4),(5,(6,(7,8))))) | 5 | 27 | 9 | 4.083333 | 0.809018 | 8 | 5040 |
| 10 | (1,((2,(3,4)),((5,6),(7,8)))) | 4 | 28 | 8 | 4.333333 | 0.714946 | 7 | 2520 |
| 11 | ((1,2),(3,((4,5),(6,(7,8))))) | 5 | 28 | 10 | 4.083333 | 0.771389 | 8 | 5040 |
| 12 | (1,((2,(3,4)),(5,(6,(7,8))))) | 5 | 29 | 11 | 3.583333 | 0.696132 | 8 | 10080 |
| 13 | (1,((2,3),((4,5),(6,(7,8))))) | 5 | 29 | 11 | 4.083333 | 0.696132 | 7 | 5040 |
| 14 | ((1,2),(3,(4,((5,6),(7,8))))) | 5 | 29 | 11 | 4.083333 | 0.752575 | 8 | 2520 |
| 15 | (1,((2,3),(4,((5,6),(7,8))))) | 5 | 30 | 12 | 4.083333 | 0.677317 | 7 | 2520 |
| 16 | ((1,2),(3,(4,(5,(6,(7,8)))))) | 6 | 30 | 14 | 3.283333 | 0.743168 | 9 | 10080 |
| 17 | (1,(2,((3,(4,5)),(6,(7,8))))) | 5 | 31 | 13 | 3.583333 | 0.639689 | 7 | 5040 |
| 18 | (1,(2,((3,4),((5,6),(7,8))))) | 5 | 31 | 13 | 4.083333 | 0.639689 | 7 | 2520 |
| 19 | (1,((2,3),(4,(5,(6,(7,8)))))) | 6 | 31 | 15 | 3.283333 | 0.667910 | 8 | 10080 |
| 20 | (1,(2,((3,4),(5,(6,(7,8)))))) | 6 | 32 | 16 | 3.283333 | 0.630282 | 8 | 10080 |
| 21 | (1,(2,(3,((4,5),(6,(7,8)))))) | 6 | 33 | 17 | 3.283333 | 0.611467 | 8 | 10080 |
| 22 | (1,(2,(3,(4,((5,6),(7,8)))))) | 6 | 34 | 18 | 3.283333 | 0.602060 | 8 | 5040 |
| 23 | (1,(2,(3,(4,(5,(6,(7,8))))))) | 7 | 35 | 21 | 2.450000 | 0.597356 | 9 | 20160 |

positions when evaluating an alignment produced automatically. We identified core columns using secondary structure information, available from the HOMSTRAD site, http://tardis.nibio.go.jp/homstrad/. Whenever the secondary structure prediction for all reference sequences agreed within one column, we considered this column as reliably aligned and designated it a core column. This methodology produces similar results to a previous study, where core columns were called if the amino acids alpha carbons was within a threshold of 0.3nm [35].

Naïvely one would assume that the TC score of a multiple alignment should be higher if only core columns are used. This is sometimes true, as for example for plantltp in the bottom-right panel of Figure (4S), where all the points are above

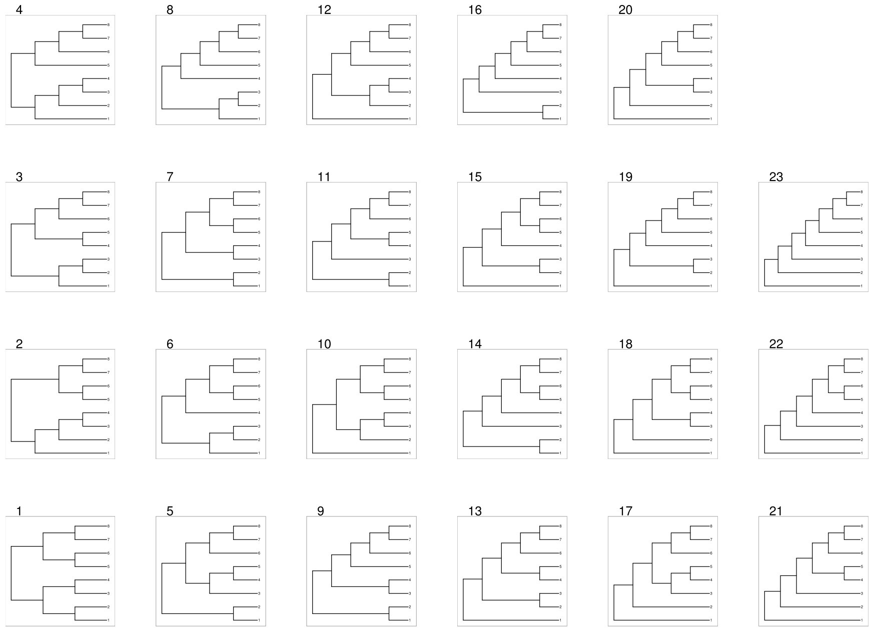


**Figure 3 Topologically distinct Trees with 8 Leaves** Dendrograms of all 23 topologically distinct trees with 8 leaves. Perfectly balanced tree (1) bottom left, perfectly chained tree (23) top of right-most column. Tree identifiers as in Table S2

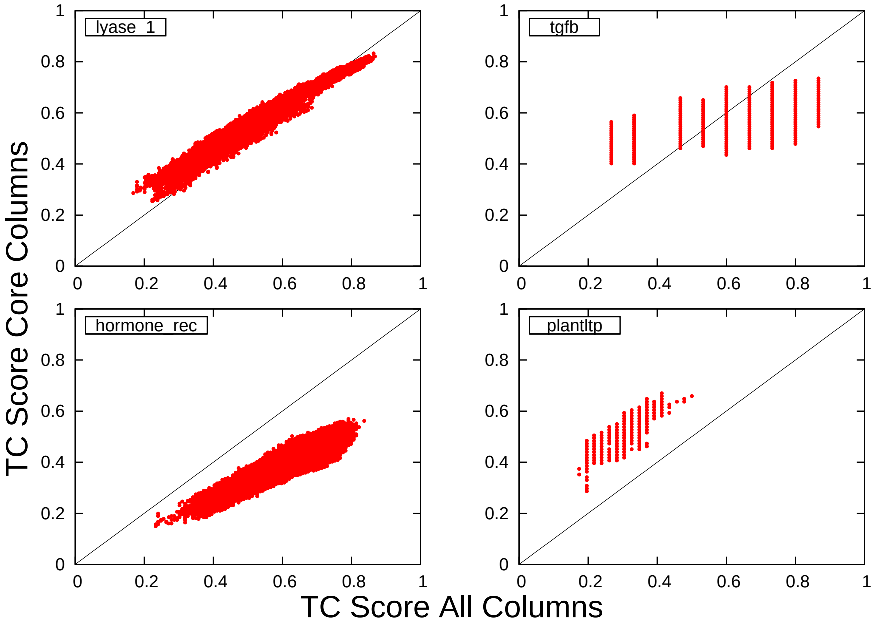


**Figure 4 Comparison of TC scores for core columns and all columns** Panels show TC scores for four example families. Along the x-axes are plotted the TC scores if all columns in the reference alignment are used; along the y-axes if only core columns are used for scoring. Each panel contains 135,135 points, some of which may lay on top of each other. Alignment were produced using Clustal Omega

the bisectrix. Two counter-examples are tgfb (top-right) and hormone_rec (bottom-left). For the latter non-core columns are apparently easy to align and the TC score

drops when these columns are removed from the scoring procedure. However, by far the most common scenario is where there is a tight correlation between core and non-core column scoring, as seen, for example for lyase_1 (top-left panel of Figure (4S).)

## (S8) Sum-of-Pairs Results

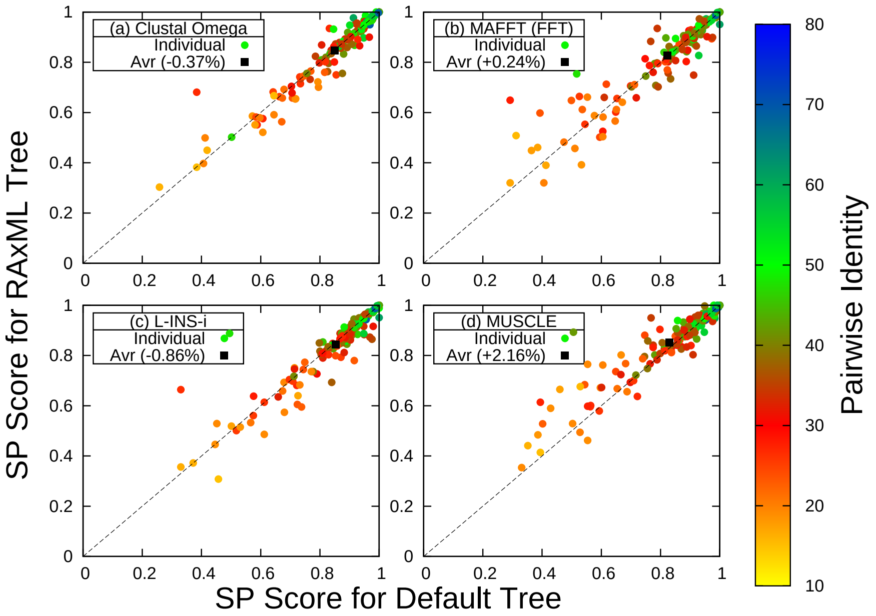


**Figure 5 Comparison of SP Scores for default tree and phylogenetic tree** SP scores for default tree along x-axis, for phylogenetic tree along y-axis for (a) Clustal Omega, (b) MAFFT FFT-NS-i, (c) L-INS-i, (d) MUSCLE. Colour dots for individual protein families. Blue and green for high percentage identity reference alignments, yellow and red for low identity. Black box for average SP score. Below dotted line default tree better than phylogenetic, above phylogenetic tree better than default tree

Figure (1) compared the individual and average results for default and phylogenetic guide-trees in terms of TC scores. Figure (5S) shows results for the same alignments but in terms of the sum-of-pairs score. The results are qualitatively similar to those for the TC score in Figure (1). However, SP scores tend to be higher than TC scores and we don't see many points in the bottom left corner. Clustal Omega and L-INS-i, again, fare slightly worse for the phylogenetic tree than for the default tree. MUSCLE, again, has slightly better results for the phylogenetic tree. MAFFT FFT-NS-i now has slightly better results for the phylogenetic tree than for the default tree, where for the TC score this was the opposite.

## (S9) Partial Results for 16 Sequences

For 8 sequences there are 23 unlabeled and 135,135 labeled trees. For 16 sequences there are 10,905 and approximately $6.2\times10^{15}$, respectively. This is not feasible, so we generated alignments for 101 topologically distinct trees with 16 leaves and reshuffled the sequences 10,000 times, leading to approximately one million alignments per family. When systematically constructing trees with 16 leaves we encountered

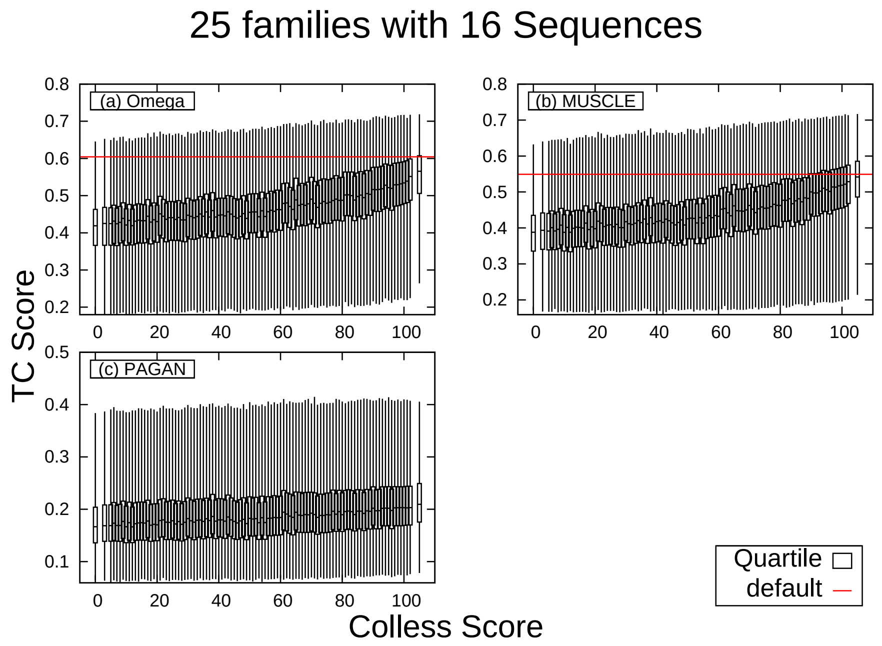


**Figure 6 Quartiles of TC scores for different tree topologies with 16 leaves** Tree topology along the x-axis, left-most box for perfectly balanced tree, right-most box for perfectly chained tree. Whiskers represent top/bottom 25% scores, band represents median score. Boxes are averages over all 25 protein families with 16 sequences. Red horizontal line average default score

101 different values of the Colless score out of a maximum of 105. There are no trees with 16 leaves and Colless scores of 1, 2, 4, 103 and 104, respectively.

We assembled 25 families with at least 12 reference sequences, padded with up to 4 non-reference Pfam sequences. In order to speed up the analysis we restricted ourselves to using Clustal Omega, MUSCLE and PAGAN. The analysis is analogous to the one for eight sequences. Results for the selected tree topologies for 16 sequences are shown in Figure (6S). We observe that on average chained trees (high Colless scores) produce better TC scores than balanced trees (small Colless scores). The results are qualitatively similar to the ones shown in Figure (6). For Clustal Omega and MUSCLE the default TC score (red line) is higher that the median of the chained result, however, for MUSCLE only just about; for the version of PAGAN that we tested there is no default.

## (S10) Proportion of Best/Worst Topologies

For each protein family we registered which guide-tree topology produced the best and the worst TC scores. If more than one tree produced the best/worst scores we assigned fractional counts to the corresponding topologies. Figure (7S) shows frequencies for the best trees for 4-8 sequences. There are many more ($2^{N-2}$ times) possible chained guide-trees than there are balanced ones. For example, for $N = 4$ sequences there are 12 chained guide-trees and 3 balanced ones. For $N = 8$ sequences there are 20,320 chained trees and 315 balanced ones. So, the pool of chained trees to draw an optimal tree from is larger than for the balanced case. For this reason we divided the raw frequencies by the number of possible trees for each topology – therefore 'frequencies' do not add up to 1.0. For all numbers of sequences analysed in this

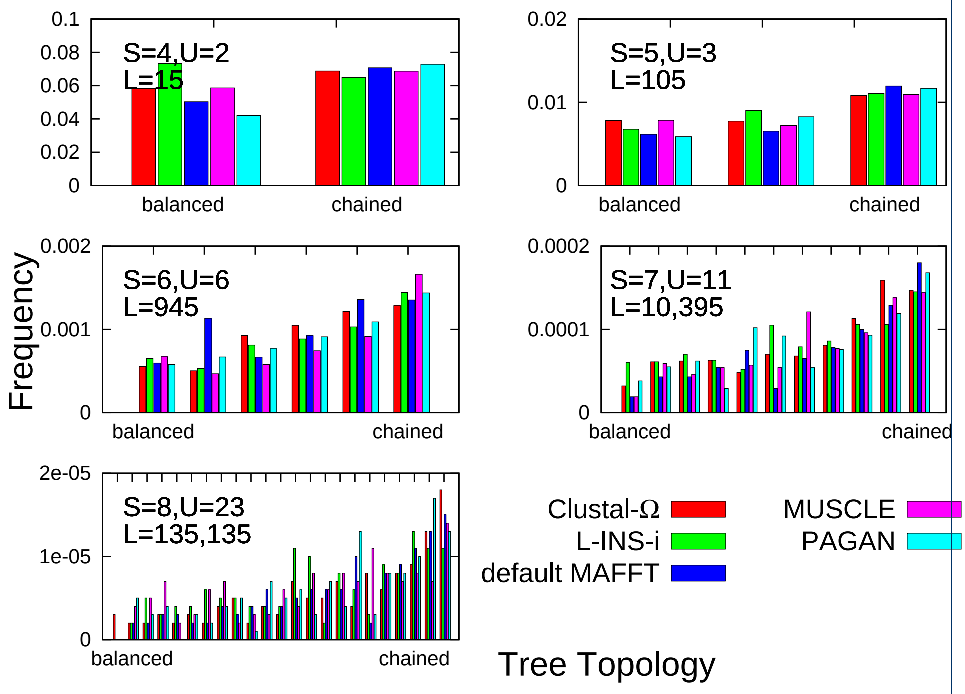


**Figure 7 Percentage how often topologies produce best score** Panels show rescaled frequencies for S=4,5,6,7,8 sequences. 'U' indicates the number of different topologies, represented by groups of histogram bars. That is, U=2 for S=4 sequences, U=3 for S=5, U=6 for S=6, U=11 for S=7 and U=23 for S=8 sequences. Left-most group of bars for perfectly balanced trees, right-most group of columns for perfectly chained trees, in-between groups as indexed in Table S2. 'L' indicates the number of distinctly labeled trees. If more than one tree produced optimum score then fractional counts were assigned. From left-to-right, results for Clustal Omega in red, L-INS-i in green, MAFFT FFT-NS-i in dark blue, MUSCLE in magenta and PAGAN in light blue

study it is always the chained guide-tree that produces most frequently the best TC scores. If the rescaling had not been performed then the histogram bars would be slanted even more towards the chained trees. This is consistent with Figure (6), in which the chained guide-tree exhibited the highest scores. This figure is only meant to provide supporting evidence of the fact that chained guide-trees potentially can be better than balanced guide-trees. In Figure (7S) we only count topologies that produce the best TC scores and discard trees that produce the second best and third best TC scores etc. This is true for all aligners.

Figure (8S) shows the opposite to Figure (7S), that is, the proportion of times a certain topology produced the worst TC score. Again, we only registered the worst and not the second of third worst scores etc. However, here the trend is less ambiguous: while the pool of possible balanced trees is much smaller than the pool of possible chained trees, they are very much over-represented when counting worst TC scores. This is true for all aligners, except for PAGAN. This suggests that PAGAN does perform sub-optimally when given a chained guide-tree. Figure (9S) is the equivalent of Figure (5), in that it displays how many families have more than a certain number of guide-trees producing the worst possible TC scores. While in Figure (5) PAGAN was particularly sensitive in picking out good guide-trees, it does not behave substantially differently in Figure (9S) in being vulnerable to bad guide-

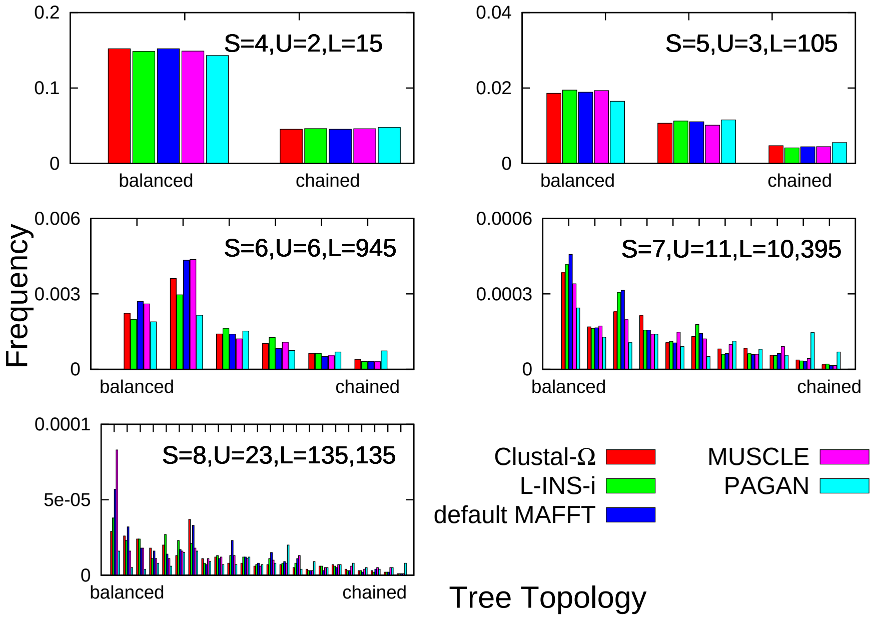


**Figure 8 Percentage how often topologies produce worst score** Panels show frequencies for S=4,5,6,7,8 sequences. 'U' indicates the number of different topologies, represented by groups of histogram bars. That is, U=2 for S=4 sequences, U=3 for S=5, U=6 for S=6, U=11 for S=7 and U=23 for S=8 sequences. Left-most group of bars for perfectly balanced trees, right-most group of columns for perfectly chained trees, in-between groups as indexed in Table S2. 'L' indicates the number of distinctly labeled trees. If more than one tree produced worst score then fractional counts were assigned From left-to-right, results for Clustal Omega in red, L-INS-i in green, MAFFT FFT-NS-i in dark blue, MUSCLE in magenta and PAGAN in light blue

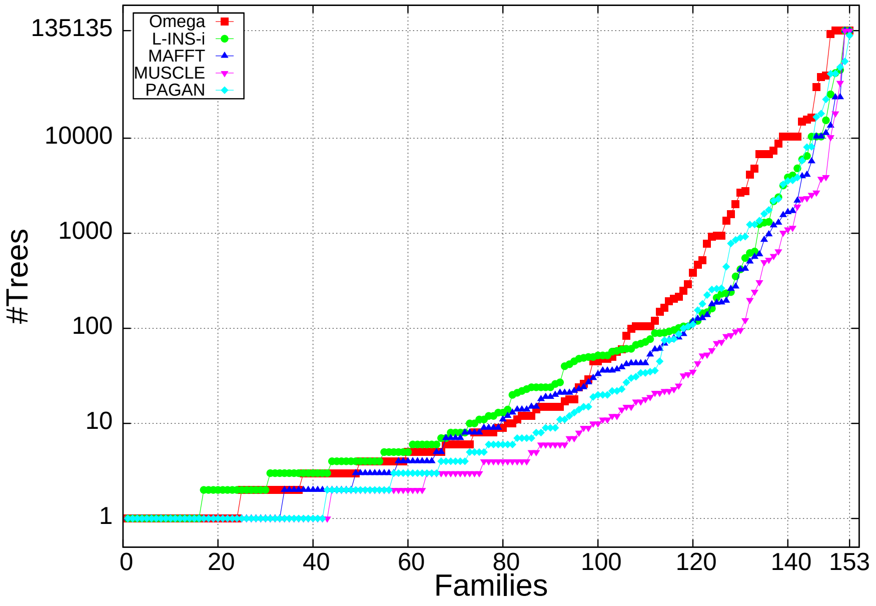


**Figure 9 Number of trees that produce worst TC score** Along the x-axis number of families with no more trees producing worst TC score than indicated along y-axis. Clustal Omega shown with red boxes, L-INS-i with green bullets, MAFFT FFT-NS-i with dark blue triangles, MUSCLE with inverted magenta triangles, PAGAN with pale blue diamonds

trees. This suggests that the accuracy of PAGAN alignments is indeed vulnerable to incorrectly labeled chained guide-trees.

## (S11) Effect of Tree Branch Lengths on Best Alignment

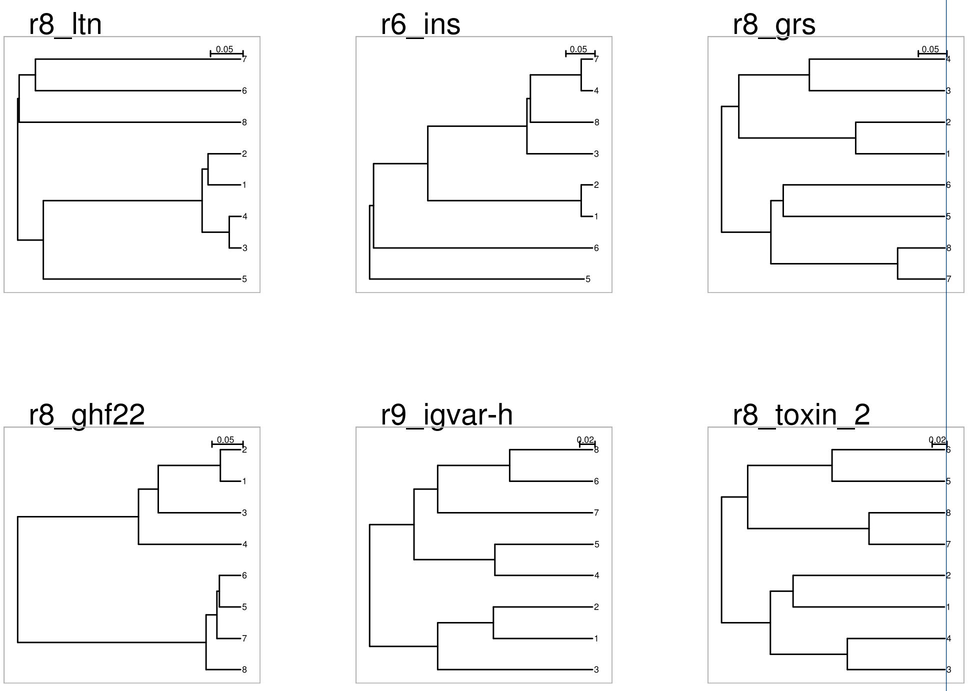


**Figure 10 Six example default trees.** Trees with a varying degree of branch length variability. These trees are identified in Figure (11S) by label.

The distances amongst individual sequences are used in constructing the default guide-trees. The distance matrix indirectly encodes the topology of the default guide-tree. In Figure (11S) we use the Colless measure of imbalance as a proxy for tree topology – perfectly balanced trees have a Colless score of 0, perfectly imbalanced/chained trees have a Colless score of 21. This figure is the same as Figure (4), where one saw that, on average, trees with small variability of branch lengths produced more balanced trees. Three examples for such low-variability / balanced trees are r9_igvar-h, r8_toxin_2 and r8_grs, which can be found in the bottom-left-hand corner of the top panel of Figure (11S).

Trees with high variability are r8_ltn, r6_ins and r8_ghf22. The default tree for r8_ghf22 is composed of two tight clusters (short branch lengths) that are loosely coupled (long branches).

However, the optimum guide-tree for r8_ghf22 is perfectly chained, as can be seen in the bottom panel of Figure (11S), where the Colless score for r8_ghf22 is 21. Apparently it is *not* necessary to align the two clusters separately. This is true for most families and can be seen by the fact that in the bottom panel most guide-tree topologies are shifted up towards the chained topology.

## (S12) Trend of Random Chained Trees vs Default

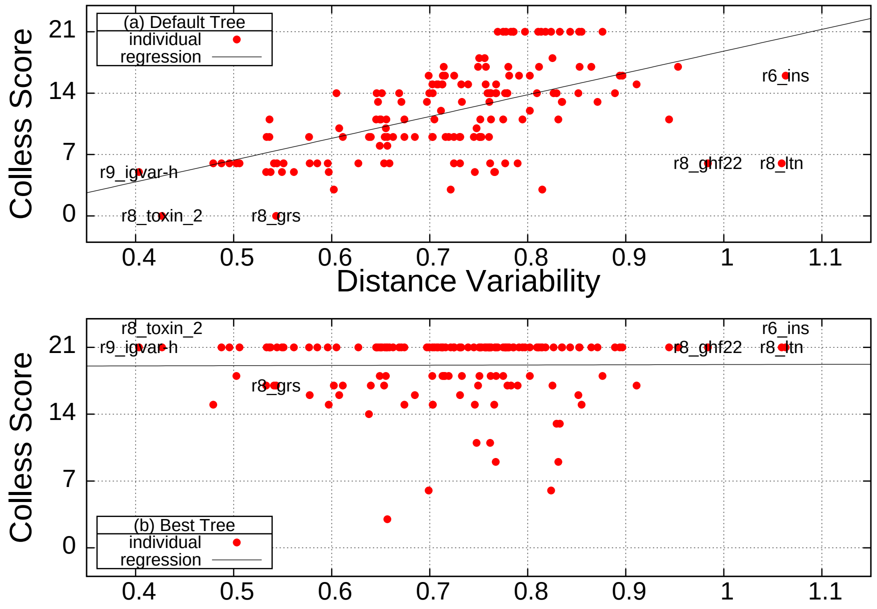


**Figure 11 Tree Topology for default and best trees as a function of tree branch length variability.** Same as Figure (4), with six example trees from Figure (10S) identified by label. Top panel shows Colless scores for *default* trees as a function of the branch length variability. Bottom panel shows Colless score for *best* guide-tree. Perfectly balanced trees have a Colless score of 0, perfectly chained trees have a score of 21.

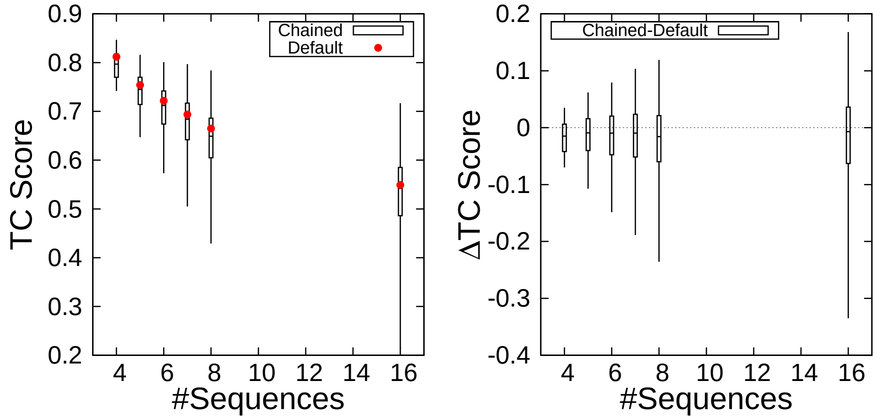


**Figure 12 Trend of TC score for chained trees using MUSCLE** (left) MUSCLE default TC scores (red bullets) and candlesticks for chained trees. Number of sequences along the x-axis. Whiskers for top/bottom quartile, band for median. (right) Quartiles of chained MUSCLE alignments relative to TC score of the default alignment

One result of this study is that chained guide-trees on average produce alignments with higher TC scores than other guide-tree topologies. The default guide-tree on average produced better TC scores than a randomly labeled chained guide-tree. This is true for all aligners and for all numbers of sequences we examined in this study (4-8,16). However, for MUSCLE this difference was only marginal. In Figure (12S) we plot the default and quartiles TC score for MUSCLE against the number of sequences. Results for 4-8 sequences are averaged over the same 153 pro-

tein families, results for 16 sequences over a reduced set of 25 families. In the left panel TC scores are decreasing with number of sequences, however, the difference between the default score and the median score appears to get smaller. For the first 5 cases (4-8 sequences) the decreasing trend is due to the general deterioration of TC scores with number of sequences, as described in [38]. TC scores for 16 sequences may be down due to the same effect or to the fact that the set of protein families is different to the ones used for 4-8 sequences. For this reason we plotted the TC scores in the right panel relative to the default score. While the spread of the whiskers grows, the median is slowly increasing from a negative value. This means that for small numbers of sequences the default MUSCLE guide-tree is on average better than a randomly labeled chained guide-tree. However, the trend is rising and a naïve extrapolation suggests that for around 35 sequences a randomly labeled chained guide-tree is on average better than the default. This is consistent with unpublished observations where for the BAliBASE 3.0 data set chained guide-trees are about as good as default guide-trees. The average sequence numbers per family for the different categories in BAliBASE 3.0 are between 7 and 63, which is of the same order of magnitude as 35, as suggested in Figure (12S).

**Availability of supporting data**
Benchmark sequences, tree topologies, utility programs and driver scripts are available as
`www.bioinf.ucd.ie/BMC-2014-treeExploration.tar.gz`

**Competing interests**
The authors declare that they have no competing interests.

**Author's contributions**
FS designed the study, selected benchmark data, generated systematic guide-trees and performed benchmarking. GMH generated the phylogenetic trees from reference alignments. DGH discussed results and helped with the manuscript. All authors wrote and approved this paper.

**Acknowledgements**
Funding was provided by Science Foundation Ireland to DGH through PI grant 11/PI/1034

**References**
1. Needleman, S., Wunsch, C.: A general method applicable to the search for similarities in the amino acid sequence of two proteins. Journal of Molecular Biology **48**(3), 443–53 (1970). doi:10.1016/0022-2836(70)90057-4
2. Feng, D., Doolittle, R.: Progressive sequence alignment as a prerequisite to correct phylogenetic trees. J Mol Evol **25**(4), 351–60 (1987)
3. Higgins, D., Bleasby, A., R, F.: Clustal v: improved software for multiple sequence alignment. Comput Appl Biosci **8**(2), 189–91 (1992). doi: 10.1093/bioinformatics/8.2.189
4. Sievers, F., Wilm, A., Dineen, D., Gibson, T., Karplus, K., Li, W., Lopez, R., McWilliam, H., Remmert, M., Söding, J., Thompson, J., Higgins, D.: Fast, scalable generation of high-quality protein multiple sequence alignments using clustal omega. Mol Syst Biol **7**(539) (2011)
5. Katoh, K., Misawa, Kuma, Miyata: Mafft: a novel method for rapid multiple sequence alignment based on fast fourier transform. Nucleic Acids Res **30**, 3059–3066 (2002)
6. Edgar, R.: Muscle: multiple sequence alignment with high accuracy and high throughput. Nucleic Acids Res **19**(32(5)), 1792–7 (2004)
7. Sneath, P., Sokal, R.: Numerical Taxonomy. The Principles and Practice of Numerical Classification. Freeman, San Francisco (1973)
8. Saitou, N., Nei, M.: The neighbor-joining method: a new method for reconstructing phylogenetic trees. Molecular Biology and Evolution **4**(4), 406–25 (1987)
9. Liu, K., Raghavan, S., Nelesen, S., Linder, C., Warnow, T.: Rapid and accurate large-scale coestimation of sequence alignments and phylogenetic trees. Science **324**(5934), 1561–4 (2009)
10. Boyce, K., Sievers, F., Higgins, D.: Simple chained guide trees give high quality protein multiple sequence alignments. PNAS **111**(29), 10556–61 (2014)
11. Barton, G., Sternberg, M.: A strategy for the rapid multiple alignment of protein sequences: Confidence levels from tertiary structure comparisons. Journal of Molecular Biology, **198**(2), 327–337 (1987)
12. Taylor, W.: A flexible method to align large numbers of biological sequences. Journal of Molecular Evolution **198**(2), 161–9 (1988)
13. Punta, M., Coggill, P., Eberhardt, R., Mistry, J., Tate, J., Boursnell, C., Pang, N., Forslund, K., Ceric, G., Clements, J., Heger, A., Holm, L., Sonnhammer, E., Eddy, S., Bateman, A., Finn, R.: The pfam protein families database. Nucleic Acids Research **40**, 290–301 (2012)

14. Löytynoja, A., Vilella, A., Goldman, N.: Accurate extension of multiple sequence alignments using a phylogeny-aware graph algorithm. Bioinformatics **28**(13), 1684–91 (2012)
15. Söding, J.: Protein homology detection by hmm–hmm comparison. Bioinformatics **21**(7), 951–60 (2004). doi: 10.1093/bioinformatics/bti125
16. Blackshields, G., Sievers, F., Shi, W., Wilm, A., Higgins, D.: Research sequence embedding for fast construction of guide trees for multiple sequence alignment. Algorithms for Molecular Biology **5:21** (2010). doi:10.1186/1748-7188-5-21
17. Notredame, C., Higgins, D., Heringa, J.: T-coffee: A novel method for fast and accurate multiple sequence alignment. JOURNAL OF MOLECULAR BIOLOGY **302**(1), 205–17 (2000). doi: 10.1006/jmbi.2000.4042
18. Mizuguchi, K., Deane, C., Blundell, T., Overington, J.: Homstrad: a database of protein structure alignments for homologous families. Protein Sci. **7**, 2469–71 (1998)
19. Darriba, D., Taboada, G., Doallo, R., Posada, D.: Prottest 3: fast selection of best-fit models of protein evolution. Bioinformatics **27**, 1164–5 (2011)
20. Akaike, H.: Information theory and an extension of the maximum likelihood principle. In: Proceedings of the 2nd International Symposium on Information Theory; Budapest, pp. 267–81 (1973)
21. Sugiura, N.: Further analysis of the data by akaike's information criterion and the finite correction. Comm. Statist. A-Theory. Meth **7**, 13–26 (1978)
22. Schwarz, G.: Estimating the dimension of a model. Ann. Statist **6**, 461–4 (1978)
23. Minin, V., Abdo, Z., Joyce, P., Sullivan, J.: Performance-based selection of likelihood models for phylogeny estimation. Syst Biol **52**, 674–83 (2003)
24. Stamatakis, A.: Raxml-vi-hpc: maximum likelihood-based phylogenetic analyses with thousands of taxa and mixed models. Bioinformatics **22**, 2688–90 (2006)
25. Felsenstein, J.: Phylip - phylogeny inference package (version 3.2). Cladistics **5**, 164–166 (1989)
26. Rogers, J.: Central moments and probability distribution of colless's coefficient of tree imbalance. Evolution **48**(6), 2026–36 (1994)
27. OEIS: Double factorial of odd numbers. http://oeis.org/A001147
28. OEIS: Number of trees with n unlabeled nodes. http://oeis.org/A000055
29. Sackin, M.: 'good' and 'bad' phenograms. Systematic Zoology **21**, 225–226 (1972)
30. Colless, D.: Phylogenetics: The theory and practice of phylogenetic systematics. Systematic Zoology **31**, 156–169 (1982)
31. Shao, K., Sokal, R.: Tree balance. Systematic Zoology **39**(3), 266–276 (1990)
32. Pavlopoulos, G., Soldatos, T., Barbosa-Silva, A., Schneider, R.: A reference guide for tree analysis and visualization. BioData Min **3**(1) (2010)
33. Larkin, M., Blackshields, G., Brown, N., Chenna, R., McGettigan, P., McWilliam, H., Valentin, F., Wallace, I., Wilm, A., Lopez, R., Thompson, J., Gibson, T., Higgins, D.: Clustal w and clustal x version 2.0. Bioinformatics **23**(21), 2947–8 (2007)
34. Biro, J.: Amino acid size, charge, hydropathy indices and matrices for protein structure analysis. Theor Biol Med Model **3**(15) (2006)
35. Blackshields, G., Wallace, I., Larkin, M., Higgins, D.: Analysis and comparison of benchmarks for multiple sequence alignment. In Silico Biology **6**(0030) (2006)
36. Robinson, D., Foulds, L.: Comparison of phylogenetic trees. Mathematical Biosciences **53**, 131–47 (1981)
37. Edgar, R.: Phylogenetic trees are not good guide trees! http://www.drive5.com/muscle/manual/guidevsphylo.html
38. Sievers, F., Dineen, D., Wilm, A., Higgins, D.: Making automated multiple alignments of very large numbers of protein sequences. Bioinformatics **29**(8), 989–95 (2013). doi: 10.1093/bioinformatics/btt093
39. Löytynoja, A., Goldman, N.: An algorithm for progressive multiple alignment of sequences with insertions. PNAS **102**, 10557–62 (2005)
40. Ogden, T., Rosenberg, M.: Multiple sequence alignment accuracy and phylogenetic inference. Syst. Biol. **55**(2), 314–28 (2006). doi: 10.1080/10635150500541730
41. Thompson, J., Koehl, P., Ripp, R., Poch, O.: Balibase 3.0: Latest developments of the multiple sequence alignment benchmark. Proteins **61**(1), 127–36 (2005). doi: 10.1002/prot.20527
